# Understanding the Value of Tourism to Seniors’ Health and Positive Aging

**DOI:** 10.3390/ijerph19031476

**Published:** 2022-01-28

**Authors:** Guanghui Qiao, Liu Ding, Keheng Xiang, Bruce Prideaux, Jinyi Xu

**Affiliations:** 1 Zheshang Research Institute, School of Tourism and Urban-Rural Planning, Zhejiang Gongshang University, Hangzhou 310018, China; qgh@mail.zjgsu.edu.cn (G.Q.); dingliu1998@163.com (L.D.); 2 School of Commerce and Trade, Zhejiang Technical Institute of Economics, Hangzhou 310018, China; keheng.xiang@connect.polyu.hk; 3 School of Business, Central Queensland University, Cairns, QLD 4870, Australia; b.prideaux@cqu.edu.au

**Keywords:** active aging, tourism value, seniors, survival analysis, narrative analysis

## Abstract

The value of tourism has been included in studies of active aging, and the existing public health implications of the physical and mental health effects of tourism among seniors are recognized as important issues. This study uses a mixed methods research approach to explore the effects of tourism value on the health and mortality risk of older adults, referred to in this paper as seniors. Survival analysis and cohort analysis are combined with the narrative analysis of in-depth interviews with eight convenience-sampled seniors to construct a narrative framework of seniors’ active aging tourism and its intrinsic drivers. The study found that the intrinsic framework of active aging tourism values for seniors has a three-stage continuum, an interaction, and orientation characteristics. There are six intrinsic key value drivers of tourism value and public health for seniors. In addition, this study identifies the personal characteristics and strengths of seniors as important influences on tourism value practices for active aging and public health. This study provides a positive psychological and behavioral research direction for existing research on the value of tourism in active aging. It provides an empirical basis for exploring the intrinsic mechanisms of tourism and public health.

## 1. Introduction

Previous research has identified aging as a problem associated with the decline and loss of physical and psychological health in later life [1]. The development of the concept of “successful aging” has inspired considerable interest in “healthy aging”, “active aging”, and “productive aging” [2] as strategies to overcome these problems. The process of active aging acknowledges the value and importance of maintaining good health for older adults while remaining in their usual environment [3]. This view has stimulated research into active aging, with emphasis on the development and maintenance of abilities and skills that lead to a greater sense of well-being. One element of well-being that has attracted increasing research is travel. To understand the travel behavior of older adults, referred to as seniors in this paper, mainstream psychological theories, such as disengagement theory, activity theory, and aging, have been developed and applied [4]. Several studies have noted that tourism positively affects seniors’ subjective well-being and level of social engagement by enhancing their self-esteem and confidence [5,6]. Given the potential for active aging to generate positive improvements in the overall health of all seniors, including their physical and mental health, activities that stimulate active aging, including tourism, have emerged as an important research area in factors related to seniors’ health.

To date, issues related to senior tourism have been dominated by market segmentation studies, with an emphasis on decision-making and motivation [4,7]. Existing studies do not adequately address the perceived tourism value and health effects of travel by seniors [8]. Although there is evidence of an association between seniors’ travel experiences and self-rated health, autonomic nervous system function, and psychological well-being, it has not been adequately established that travel experiences are associated with positive improvements in seniors’ physical and mental health, and relationship survival, either at an individual level or at a national level [9].

Negative cultural stereotypes experienced by seniors may hinder the positive impacts of active aging. Fernández-Ballesteros and Sánchez-Izquierd [10], for example, reported that, in many European countries, widespread negative cultural stereotypes of seniors, including the view that seniors may have high enthusiasm but low ability in relation to active aging, poses obstacles and limitations to the practice of active aging in this age group. However, in recent decades, seniors have become more actively engaged in tourism than in the past [11], leading to positive emotions of transcendence, improved mental state, enhanced subjective well-being, and improved health outcomes in the overall population of seniors [12]. For the purposes of this paper, issues related to the overall health of the national cohort of seniors are referred to as public health. The lack of evidence in existing studies to support the association between seniors’ travel experiences and public health has prompted enquiries into the intrinsic mechanisms linking seniors’ travel values to their physical and mental health, public health, and active aging. This study proposes three core research questions on the topic of seniors’ public health through the lens of active aging. First, what is the relevance of seniors’ tourism behavior to their physical, mental, and public health? How does the health effect of tourism value unfold? Second, how is the internal framework of the positive aging narrative of seniors constructed? How is it responsive to the efficacy of seniors’ public health? Third, how are the key value drivers inherent in tourism for seniors and public health for older adults constituted, and how are the value practices of active aging tourism explored? The answers to these research questions will assist in the identification of the intrinsic linkages and mechanisms of tourism value and public health that promote active aging.

The potential value of this study is the use of the developmental cycle of active aging as a lens to explore the value of tourism, as well as the intrinsic linkages between tourism and seniors’ public health. It examines the positive psychological adjustment and personal trait strengths of seniors that promote positive mental health adaptation pathways, and identifies a positive psychological dimension that can contribute to a deeper understanding of the existing relationships between tourism and the public health of seniors. This was achieved by focusing on the public health effects of tourism value and active aging to provide an empirical basis for further clarification of the mechanisms underlying older adults’ travel behavior and active aging.

## 2. Literature Review

### 2.1. Senior Tourism

In this research, the study population are described as seniors, in preference to older adults. The literature has yet to offer a universally agreed definition of senior tourists, with definitions ranging from participants in tourism activities who are over the age of 50, to studies that define senior tourists as being over 60, or even 65 [13]. Most studies show that seniors’ motivation for travel is mainly the pursuit of happiness, escape from daily life, nostalgia, and lifelong learning, but they are also limited by factors such as physical condition and family responsibilities [14,15].

Issues related to senior tourism market segmentation have been widely researched, with the main segments described variously as new seniors, young seniors, affluent seniors, and retired baby boomers [16]. Senior tourism market research has also investigated issues such as how the tourism market responds to the “silver-haired” segment. However, few in-depth studies have addressed the psychology and behavior of older travelers. Studies have shown that senior travelers are mainly interested in leisure, and are not aware of the potential health benefits of travel [17]. Other studies have focused on the exploring the mechanisms and factors that influence tourism well-being in seniors. Milman [18], for example, explored the effect of tourism on the level of well-being of older travelers, and found that the educational, recreational, aesthetic, and escape aspects of the tourism experience economy contributed to the well-being of older travelers. Chen et al. [19] proposed that learned helplessness is an important psychological construct that affects seniors’ subjective well-being. In existing studies on the travel well-being of seniors, positive aging and positive psychological perceptions have emerged as major areas of research. The literature also includes a relatively large volume of research on factors influencing seniors’ travel behavior. In an empirical study, Jang et al. [20] found that an individual’s emotional state and motivation to travel can affect the travel willingness of older Taiwanese adults. Kazeminia et al. [21] found that gender, self-perceived economic status, and disposable time limit the frequency of travel among seniors. The correlation between travel and the physical and mental health of seniors was also noted by Hunter-Jones and Blackburn [22], who found that vacations induce subjective feelings of relaxation and well-being in seniors, and, in some instances, lead to reported improvements in certain chronic diseases, such as asthma and arthritis. This study will identify the mechanisms that govern the intrinsic association between travel and the physical and mental health of seniors, following the direction of the current research, and building a framework to provide a theoretical basis for investigating aspects of seniors’ tourism activities.

### 2.2. Tourism Value and Older Adult Travelers

Existing studies on the association between tourism value and senior travelers have identified a number of areas of interest, including the value elements, such as social value, physical and mental value, active aging, and quality of life value, that tourism brings to seniors. Qiao et al. [6,23] highlighted the community value of tourism in forming and developing new relationships for seniors, and creating opportunities for seniors to interact with others [24]. In terms of physical and mental value, Fleischer and Pizam [25] emphasized the positive effects of tourism on seniors’ physical and mental health, with nostalgic experiences and physical exercise being examples of this value. Lee and Tideswell [26] and Kim and Woo [4] provided the interpretation of this value. Other studies have found that active aging, as a value pursuit, has significant benefits for the social interactions and physical and psychological health of seniors [27], and that tourism is an important part of their post-retirement life, often serving as an adaptive strategy to help retired people cope with normal aging [28].

Active aging as an important element in achieving positive value outcomes of seniors’ tourism, and has been explored in a number of recent studies. Yoon et al. [29] identified healthy aging for sustainable development as a value output, and argued that seniors’ participation in meaningful leisure activities was important for sustainable aging through education, and encouraging older adults to participate in leisure activities to enable them to acquire leisure skills, knowledge, and motivation. Zhang et al. [30], on the other hand, proposed health-oriented seasonal tourism as an innovation in the value approach to active aging, highlighting the relationship between tourism venues and the existence of active aging among seniors. The role of subjective well-being in the value of tourism for seniors was observed in a study on Korean seniors [31], and Canham et al. [32] emphasized the functional importance of tourism for the satisfaction of seniors’ psychosocial needs in the process of active aging. Zhang et al. [30], in a study of social and physical landscapes, postured that the positive impact of restorative experiences on health perceptions of active aging confirms the close relationship between the value of tourism and leisure for seniors. On this basis, a number of studies have framed active aging as an important element of seniors’ physical, tourism, leisure, and subjective well-being. Further, the concept of sense of coherence proposed by Antonovsky [33] has been used as the basis for a tourism and leisure and active aging theoretical framework, which has been used to explore the factors that play a role in maintaining a healthy state among the elderly.

Currently, the literature on the value of tourism among elderly people is largely oriented toward positive psychology and positive relationship construction, and provides a theoretical reference for the exploration of the key influencing factors of the value of tourism for seniors in this study. However, the empirical arguments of existing studies on the value of tourism for seniors, and the intrinsic mechanism of the process of active aging, are insufficient to explain how tourism assists in active aging, or how tourism can promote improvements to physical and mental health. The lack of research of this type provides a strong justification for the research described in this paper.

### 2.3. Applications of the Survival Analysis Model in Seniors Tourism Research

The survival analysis model, which has been employed to explain the length of tourists’ stay at a destination [34], is a statistical technique used to analyze longitudinal data on the occurrence of events such as death in biological organisms, and failure in mechanical systems, and is an appropriate statistical method to analyze time-event type data [35,36]. The use of this model in senior tourism research is limited, and existing survival analysis models in senior tourism research are mainly focused on two topics: the length of stay in tourist destinations, and tourism decision-making. Barros et al. [37] used a survival model to analyze the vacation stay of older tourists, and found that age influenced length of stay. Ashrafi et al. [38], in their application of survival analysis theory on tourism in Norway, found that younger tourists with fewer budget constraints had a higher probability of survival than low spending older tourists. Tourists who left Norway for the purpose of visiting family and friends had a lower risk of leaving the country than those who left for other purposes. Survival rates were higher for those choosing camping as their main form of accommodation, and road transport as their preferred mode of transportation. In terms of decision-making, Li et al. [39] used a discrete-continuous model framework to simulate tourist behavior in choosing a travel season (choice model), and the associated travel length of stay (accelerated lapse time model). They established a correlation between the two travel decisions conditional on covariates, finding that seniors’ leisure and travel activities were associated with longer air travel and higher income, whereas higher income was associated with a shorter length of stay. Building on the results of previous research (Ashrafi et al. [38] and Li et al. [39]), this study explored the mechanisms underlying the health effects and mortality risks of travel by seniors using an applied survival analysis model oriented to a longitudinal data cohort on time-to-event orientation.

### 2.4. Theoretical Gap

Existing research on senior tourism has mainly focused on the “silver-hair market”, with destination marketing as the main topic of research. Research on tourism value segmentation has also explored micro-value influencing factors, such as community, mind and body, and socialization [26]. Collectively, these have formed a systematic research path. However, few studies have explored the intrinsic associations and mechanisms that explain how tourism can yield positive health and active aging outcomes for seniors. In active aging, attention should be paid to the micro tourism behaviors and the psychology of seniors. Issues that need to be addressed include: How can positive tourism behaviors and positive psychology construct and foster active aging? What is the basis of the real tourism value transformation path for seniors? How does survival analysis theory provide an explanatory path for the intrinsic relevance of tourism and active aging? This study will explore these issues by combining survival analysis theory and tourism value theory.

## 3. Methods

### 3.1. Design and Participants

This study applied a mixed methods approach, using quantitative data to determine trends and relationships, and qualitative research to explain the mechanisms and reasons behind these trends. The study design was divided into two parts. Study I explored the effects of travel behavior on health risk among seniors by using data representative of Chinese adults aged 65 years and older. The study was based on the survival analysis approach with strong causal validity. Study II recruited respondents through convenience sampling in a snowball format. A total of eight respondents (four males and four females) were interviewed. All respondents were retired, none had underlying medical conditions, and all had some travel experience. Appendix A shows the interview outline. The data for study II were collected over a one-month period in late 2021.

Convenience sampling, also known as availability sampling, is a non-probability sampling method that relies on collecting data from members of a population willing to participate in a study [40]. For the purposes of this study, convenience sampling assisted in the generation of research hypotheses, was able to be collected over a short period of time, and was inexpensive. This study adopted a snowball sampling approach based on referrals from older adult respondents.

### 3.2. Data Analysis

In the Stage I analysis, Stata 16.0 software was used to explore the effect of travel on the risk of death among seniors, based on survival analysis. The study used the Kaplan–Meier method to plot survival curves; and a Log-rank χ^2^ test and a Cox proportional risk model to describe and statistically test for the risk of death. The results were considered significant at two-sided *p* < 0.05. Kaplan–Meier curves were used to show the survival time of seniors. Log-rank χ^2^ was used to test whether the difference in mortality risk was significant among seniors with different basic demographic characteristics, health status, and lifestyle habits. The Cox proportional risk model was used to calculate the risk ratio. The Kaplan–Meier analysis method and the Cox proportional risk model are nonparametric methods/models that do not require any assumptions about the distribution of the dependent variable, and are not affected by the shape of the baseline risk function. These methods are the most commonly used in survival analysis studies. However, the proportional risk assumptions need to be satisfied for analysis using Cox regression models. If they are not fully satisfied, they may reduce the accuracy of the estimation of parameter values. Before conducting formal analysis, the study conducted a Schoenfeld residual method test. The results showed that the proportional risk assumptions were satisfied, and that the Cox regression models can be applied to analyze the effect of tourism on the risk of death in seniors. The mathematical Cox proportional risk model was expressed as follows:

$$\ln h\left(t_{i}|x_{i}\right)=\ln h_{0}\left(t_{j}\right)+\beta_{i}C_{i}^{\;}$$

where $h\left(t_{i}|x_{i}\right)$ is the risk function with influence factor *x* at time *t*, $\ln h_{0}\left(t_{j}\right)$ denotes the baseline hazard, and *β* is the coefficient matrix of covariate $C_{i}^{\;}$.

**Figure 1:**
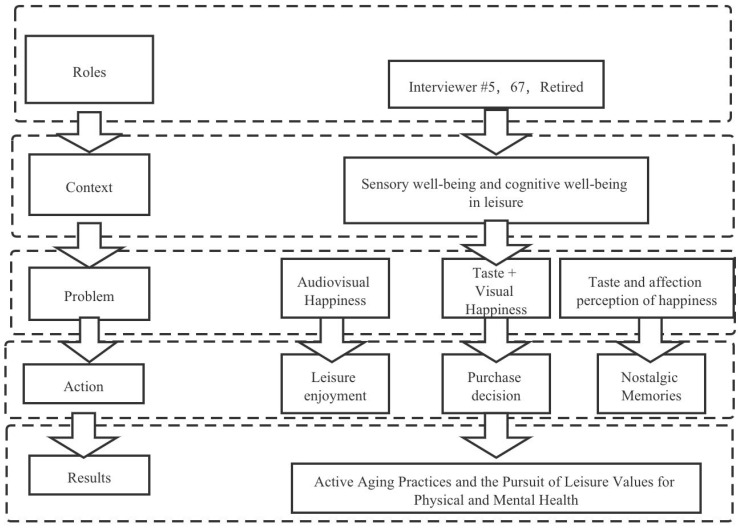
Example of respondent narrative analysis coding of R5.

In stage II, interviewee transcripts were manually coded and analyzed. A five-factor problem-solving approach proposed by Yussen and Ozcan [41], and based on role, situation, problem, action, and outcome, was employed to analyze the narratives. This approach enabled the development of a narrative structural map based on the interview transcripts [41]. A case study of the narrative coding process is shown in Figure 1. Study II grouped findings into three core topics.

### 3.3. Ethics and Reflexivity

The researcher explained the purposeful nature of the study to the senior respondents, reading the study information sheet and informed consent form aloud to participants. All interview notes were treated as confidential, and not disclosed to third parties. The researcher was conscious of the potential for the emergence of a researcher-dominated unequal status and power relationship with the respondents. To mitigate this possibility, the researcher conducting the interviews undertook ongoing reflection to ensure that an unequal power and status did not emerge. To protect the privacy and personal information, interviewees were assigned anonymous code numbers from R1 to R8. All interviewees were made aware of the privacy protection of this study prior to the interviews.

## 4. Results

### 4.1. Study I Quantitative Findings

Study I explored the effects of travel behavior on health risk among seniors using data representative of Chinese adults aged 65 years and older.

#### 4.1.1. Data Sources

Data for study 1 were extracted from the Chinese Longitudinal Healthy Longevity Survey (CLHLS) for the years between 2008 to 2018. Four sets of longitudinal data were obtained, and three prospective cohorts were constructed. The CLHLS survey is representative of the Chinese national senior population, with data covering more than 860 counties, county-level cities, or districts in 23 provinces, cities, and autonomous regions.

The study divided the longitudinal data into three cohorts of ten years, namely “2008–2018”, “2011–2018”, and “2014–2018”, to analyze the immediate and long-term effects of tourism on mortality among seniors. The study population in each cohort were adults aged ≥65 years in the base period survey. Study subjects aged ≥105 years were excluded because, according to the data quality assessment of the CLHLS survey project team, the information for the very elderly may be biased [42]. After cleaning the key variable data, and removing missing values, three cohorts were included in the analysis for the three periods (2004–2008; 2008–2012; 2012–2016).

#### 4.1.2. Variable Measurement

The independent variables adopted for this study were travel participation and number of trips. Combined with the CLHLS questionnaire, the specific number of trips within the last two years were used as variables of interest for the study. A code of 1 means that seniors had traveled within the last two years; otherwise, the code is 0. This study also analyzed the total number of trips as a key independent variable to analyze the health effects of travel.

The dependent variable adopted for this study was risk of death (i.e., the number of surviving months between the baseline survey time point and the date of death for the respective older adult). If a person died during the observation period, the length of survival was based on the number of months that had elapsed between the first visit and the time of death. If a person died during the observation period, the survival time was measured as the duration from the first survey to the most recent survey. The survival time of a person who had not died at the time of the last survey was measured from the first survey to the last survey. In the sample, persons who died were defined as “events”, and those who died during the second follow-up visit and those who were alive at the last survey were defined as “deletions”.

The control variables adopted for this study were based on existing studies [43], and included the risk of death based on demographic characteristics, such as health status and lifestyle habits of the baseline survey of the respective cohort. Basic demographic characteristics included age (years), sex (male = 1), whether they were married (yes = 1), type of residence (urban = 1), education (educated = 1), log household income ($), and whether they lived with family (yes = 1). Health status included whether they could take care of themselves in daily activities (no = 1), and whether they had been diagnosed with chronic conditions (yes = 1). Lifestyle habits included whether they exercised regularly (yes = 1), whether they drank alcohol regularly (yes = 1), and whether they smoked (yes = 1). Table 1 shows the basic profile of the control variables.

#### 4.1.3. Descriptive Analysis

Table 1 shows the detailed demographic information of the sample population. It shows that the three cohort populations most likely to travel were more likely to be male rather than female, and younger in age. In addition, older adults who traveled more had the following characteristics: a high education level, lived in urban areas, had a high annual household income, did not have a chronic disease, did not smoke, reported low alcohol consumption, and exercised regularly.

#### 4.1.4. Cox Proportional Risk Model Results

Table 2 presents the results of the f basic descriptive study and univariate tests used to identify the effect of travel on the risk of death. Cox proportional risk models based on prospective cohort data constructed from a ten-year follow-up survey, and based on three time cohorts (2008–2018 cohort, 2011–2018 cohort, and 2014–2018 cohort), yielded a total of three cohort models and three models using the number of trips as the independent variable.

As seen in Models 1-1 and 1-2, in Table 2, the results of the analysis of the ten-year longitudinal data suggest that travel has a beneficial long-term health effect. Travel within the last two years reduced the risk of death by 36.6% (HR = 0.634, 95% CI = 0.551–0.728), and had a similar “dose–response” effect. The more trips taken, the lower the risk of death. An increase in the number of trips was associated with a 13.6% reduction in the risk of death (HR = 0.864, 95% CI = 0.808–0.925).

To test the accuracy of the findings, this study constructed new cohorts for analysis using the eight-year and four-year survey data tracked by CLHLS 2011–2018 and CLHLS 2014–2018, respectively. As seen in Models 2-1 and 2-2, the analysis of the eight-year follow-up data showed that the death rate among seniors with regular travel experience slowed by 36% (HR = 0.640, 95% CI = 0.543–0.755). Their risk of death was 0.64 times lower than that of seniors without travel experience, and an increase in the number of trips was associated with a 10.7% reduction in the risk of death (HR = 0.893, 95% CI = 0.842–0.948).

In addition, analysis of the four-year follow-up data from Models 3-1 and 3-2 showed that travel reduced the risk of death by 31.3% (HR = 0.687, 95% CI = 0.526–0.897), and an increase in the number of trips slowed the rate of death by 10.6% (HR = 0.894, 95% CI0.804 = –0.995).

Taken together, the calculated values for the three cohorts were similar, and the results were robust, with all data showing that undertaking regular and frequent travel significantly reduced the risk of death in seniors.

### 4.2. Study II Findings

The qualitative research in study II discusses the mechanisms and factors that highlight how participation in tourism may have a positive impact on seniors’ health through active aging.

The cohort analysis undertaken in Study I indicated that persons with a higher number of trips demonstrated a low risk of death, indicating a causal relationship between tourism and positive long-term health outcomes. However, the underlying mechanisms of how tourism affects the physical and mental health of seniors at the micro-psychological and behavioral levels, and the potential association of tourism with active aging, were not clear. Study II was undertaken to explore the relevance of tourism values on seniors’ physical and mental health, and active aging. Based on the narratives of the eight participating seniors, this study identified a number of factors that constitute the key value elements that influence active aging, and the mechanisms for realizing seniors’ tourism value practices.

#### 4.2.1. Intrinsic Framework of Staged Tourism Value Narratives of Active Aging

**Figure 2:**
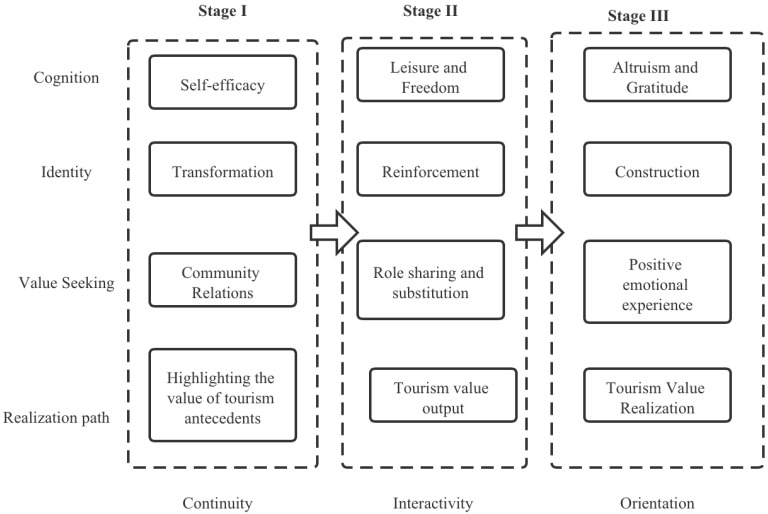
The intrinsic framework of the active aging stage tourism value narrative.

The eight seniors who participated in this study all maintained a reasonable frequency of travel. The oldest participant was aged 73 and the youngest was aged 65. All were retired and free from underlying illnesses. Appendix B shows the respondents’ basic information. This study used narrative coding of the interview transcripts to develop a narrative framework of the three stages of travel values (see Figure 2). Respondents’ aging is a continuous process of accepting self, recognizing self, and perpetuating self. This process involves different cognitive patterns, identity mechanisms, value-seeking approaches, and realization paths at each stage of retirement. In the first stage of travel value, respondents had just retired and needed to adapt to having more leisure time. In this stage, respondents’ self-efficacy was enhanced, as they completed their adaptation to the pace of life after retirement. They achieve a shift in identity, reduced isolation and loneliness, maintained their pre-retirement community relationships, and continued the value role brought about by their pre-retirement travels, as described by R1 and R5.

*Finally, I have retired but I still want to enjoy life. I retired first, and my husband is still working. Every day, I awake to a life that seems empty. I miss all the outdoor activities, and my friends feel the same. Suddenly, I understand that I have a lot of time for my favorite outdoor sports and to engage in long-distance travel. I no longer need to worry about my children. They have their own families. My husband is still working.*

(R1, 62, retired for 3 years)

*It took me a long time to adapt after retirement. Even still, I sometimes feel abandoned by society. My children have grown up and are busy at work. Before I retired, I traveled quite frequently, and I still need to be mentally prepared to maintain a high frequency of travel after retirement. Some of my friends who used to travel together have minor illnesses, and some have chronic conditions, so they can no longer engage in long-term travel.*

(R5, 67, retired for 5 years)

In the second stage of travel value, respondents talked about their deepening understanding of leisure and freedom. In adapting to post-retirement life, the respondents entered the second stage, centered around strengthening their own identification with travel and health. Some had taken on the role of group travel leader. They had positive experiences of arranging travel itineraries and organizing participation. They were able to coordinate the whole trip and share the value of their travel experience with a group of friends, as described by R2.

*I retired early and became part of the retired community. After the post-retirement psychological struggle and emotional transformation, I realized that I was still very influential in our outdoor circle. I started to organize short outdoor trips. Anyway, with so much time to spare, I began to persuade my friends to actively participate in the activities I organized. We went camping at Qingshan Lake and hiking at Changle Forest, places that were not too far away. In these short outdoor trips, we could relax, follow our interests, and have a clearer understanding of how to plan retirement life.*

(R2, 62, retired for 5 years)

After experiencing the interactions that typified the second stage, the importance of goal orientation became clear in the third stage. Analysis of the transcripts indicated that, by the third stage, respondents had begun to construct new identities, and explore new travel patterns and activities to maintain the positive emotional experiences that translate the value of travel into daily life. Some of the interviewees in this stage had organized and operated tours in the second stage, and began to engage more frequently in altruistic behavior. Gratitude and altruism become the cognitive characteristics of this stage in terms of positive group relationship building through travel. These characteristics also allowed the interviewees to gradually construct a group identity based on leisure and tourism, which became the basis for realizing tourism value. The interview transcripts of R3 and R5 present the salient features of this stage.

*Since I retired, for the past 10 years, I have organized big and small, short and long trips, and I am aware of my emotional stability. Through travel, I have slowly resolved the conflicts and contradictions in my family. Travel is like life, filled with ups and downs.*

(R3, 69, retired 10 years)

*I have several interest circles that I joined after I retired. One of them is called Southeast Asia Circle, which is all about island trips to Indonesia, Okinawa, Japan, Thailand, and so on. I divorced my husband many years ago, and I live alone. I travel to cultivate my own interests and to help others to resolve negative emotions and psychological issues. I gained a lot in the process. My shoulder and neck problems were alleviated by the non-stop physical exertion involved in my travels. I also organized the exploration of different destinations for others in similar circumstances to my own. In this way, my circle of friends could feel the charm of travel.*

(R5, 67, retired for 10 years)

Respondents highlighted different stages of tourism value in their aging narratives, interpreting, in turn, the continuity, interaction, and orientation of the aging tourism value narrative. The characteristics and internal mechanisms of the three stages provide a time-series perspective interpretation to reveal the impact of tourism value on active aging, and public physical and mental health.

#### 4.2.2. Key Driving Value Elements of Active Aging

**Figure 3:**
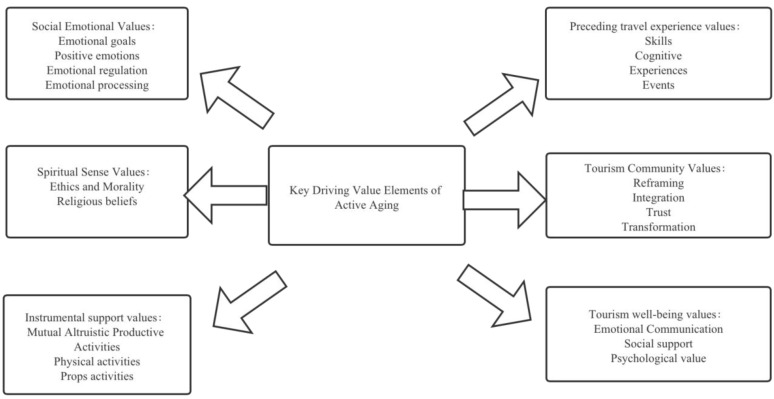
Key driving value elements of active aging.

What drives respondents’ value-seeking in active aging? What role does tourism value play in the process of active aging? This study responded to these issues by uncovering the key driving value elements in respondents’ narratives at the level of tourism value output. Figure 3 shows the specific driving value element map. Respondents proposed emotional value as the primary factor, where emotions dominate life goals and daily life progress. Tourism constitutes a leisure space that is different from daily life, where respondents are able to regularly switch from the contradictions and conflicts of their daily routine by substituting positive emotions gained through travel. The second value is that of spiritual perception. Most of the respondents have religious beliefs, which appeared to provide them with a value output that helped them in dealing with people and daily decisions. These respondents reported that they undertook regular trips associated with their religious beliefs. Those with more practical values engaged in shuffling daily space and leisure space. In the process of constantly switching between spaces, the respondents realized the output of self-value through mutual help and altruism. The respondents’ experience in these spaces provided instrumental support value for the critical group drive aspect of active aging. The narratives of interviewees R8 and R1 highlight these inferences.

*I have a bad relationship with my family and little contact with my children, but I often travel to Buddhist travel places to keep my inner peace and emotional calmness. I have my own plan to cope with my life after retirement, which is to constantly maintain a positive state of mind, not to have big ups and downs. This is a good way to travel to different travel places according to how I feel.*

(R8, 65, retired 6 years)

*My husband is still on a career path. My daughter is married. I worked in a textile factory and retired early after major surgery. I took up outdoor sports to help my recovery. When I was young, I was argumentative and headstrong. After retirement, I began to believe in Buddhism. Every month I would go to Buddhist travel places to eat vegetarian food, to chant, and to become calm. I also organized a lot of outdoor activities. In an outdoor circle of friends, everyone can be a leader and explore different places with others. Now, I am in good physical condition. My long-term outdoor activities have made me stronger. I can eat and sleep well. I think that is enough.*

(R1, 63, retired 3 years)

Respondents embodied the value of antecedent travel experiences, the value of belonging to a travel community, and the value of travel well-being in relation to travel value. They generally believed that previous travel experiences and the skills they gained have a significant impact on their life. They cultivated specific travel cognition and experiences that can be used as instrumental behaviors to plan and implement aging travel practices. Community value, as the key to sociability, had a major impact on respondents’ reconfiguration of daily living space and travel space. These spaces now have greater significance, and respondents can maintain an active and positive attitude and state of mind in the community, which can be transformed into their own value-seeking needs. Eventually, the goal of travel well-being value is able to be achieved. The result is positive emotional communication through regular travel and leisure, whereby social support is actively sought and achieved, and every aspect of the aging process is improved and optimized by the psychological capital of a positive mindset.

#### 4.2.3. Positive Aging and the Mechanism of Realizing Tourism Value: Tourism Value Practices of Older Adults

**Figure 4:**
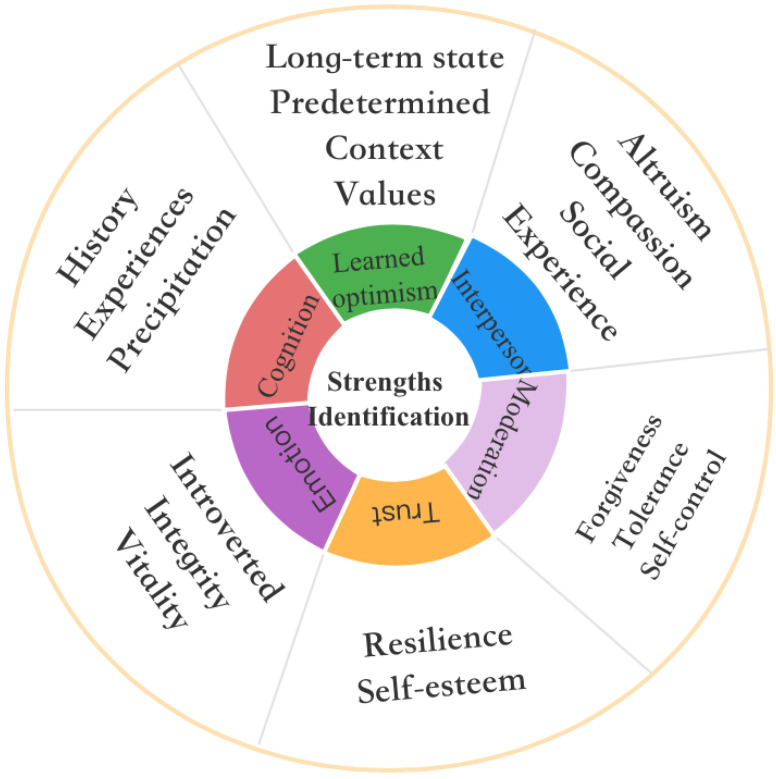
Active aging and tourism value realization mechanism: elderly tourism value practices.

This study found six strengths that promote active aging by identifying a supporting role for seniors’ tourism value practices, and the key factors that explain the mechanisms of tourism value (see Figure 4). The interviews revealed that respondents consciously or unconsciously transformed tourism values through their own strengths and attributes, enabling them to contribute to their own mindset of active aging and the construction of positive relationships. At the level of cognitive strengths, respondents came from different work-experience backgrounds. They had experiences and skills that translate into strengths in their daily lives. These emotional strengths are critical to the practical application of this combination of strengths. Respondents’ introversion and integrity, dialectic outlook on life, and mature decision-making skills allows them to make more accurate decisions during travel. At the level of belief strengths, respondents demonstrated resilience in traversing the space of travel and the space of everyday life. Their confidence in resolving setbacks and difficulties highlighted the role of self-esteem. Respondents also demonstrated an ability to exert self-control. They could balance their mind and body, forgive others, and maintain and construct balanced relationships. They were physically and mentally driven, and they were tolerant of a certain level of negative events and emotions. At the level of interpersonal strengths in the aging process, respondents were able to enjoy active and positive status by maintaining community relationships. Altruism, kindness, and social experience motivated the respondents to achieve the optimization and iteration of interpersonal relationships. Finally, this study unexpectedly found that respondents who participate in frequent travel experience have become more optimistic in outlook, and are able to actively and positively think outside their original closed environment when facing setbacks and difficulties. They are also able to use pre-defined optimistic situations to enhance their long-term mental state, and can realize the internalization and transformation of healthy values. In short, respondents’ strength identification drives the development of the potential impact of tourism values on active aging. The identification of seniors’ tourism value practices, and the value practices themselves, contribute to the construction and extension of active aging.

## 5. Discussion

The quantitative findings of this study show that the experience of traveling, and the number of trips undertaken, significantly reduce the risk of death among seniors. The cohort analysis undertaken as part of the studies’ survival analysis includes seniors who were highly educated, live in urban households, do not have chronic diseases, and had sufficient disposable income for travel, implying that tourism has long-term health effects. The qualitative findings of this study have identified the underlying mechanisms and relevant correlation arguments to underpin the quantitative results. The key finding of this study is that the health effect of tourism value for seniors lies in the process of active aging. Based on this finding, continuity, interaction, and orientation can be identified as the key issues that govern the extent to which tourism can play a positive role in active aging. Social emotion, spiritual perception, and instrumental support thus become the key driving elements of active aging. The identification of key personal traits and strengths of seniors explains tourism value and the health effect, and provides a strong analytical basis for explaining the health effects of tourism on seniors, translating the effectiveness of active aging and tourism value practices. These findings echo previous research on active aging, including Tung and Ritchie’s [44] five aspects of the positive meaning of travel for older adults. These aspects were identified as identity formation, family milestones, relationship development, nostalgia reenactment, and freedom seeking, confirming that travel helps to improve older adults’ physical and mental health to increase life expectancy, and to enhance the process of self-enrichment and the completion of self-identity [45]. The findings about tourism value orientation of active aging are also consistent with the positive meaning of well-being in later life, and the pursuit of physical and mental health values identified in previous studies [46]. In particular, positive social emotions and instrumental support have been shown to enhance seniors’ travel autonomy, their perceptions of travel value [20], and personal traits and strengths identification. Collectively, these factors explain the facilitators of seniors’ travel value attainment [14] (see Figure 3).

The findings make several contributions to theory in relation to the role that a positive psychology perspective can play in supporting active aging. The empirical analysis of tourism value and its health effects on seniors utilized the survival analysis model with a time cohort and event type, highlighting the advantages of using tourism value in senior research. This study links the theoretical value of this correlation analysis to the role of positive psychology, deepening and extending the boundaries and conditions of the use of this theory. It thus provides a theoretical framework for identifying positive relationships and the value of positive psychology and emotions in senior research. Unlike the previous use of learned helplessness theory to explore the impact on seniors’ travel health [19], this study combined a positive psychology perspective and tourism value theory to provide a practical and realistic exploration of active aging. It provides a strong basis for identifying the mechanisms for the integration of positive aging as a tourism value theory and a positive psychology theory, providing a clear direction for future research in these areas. The findings also contribute to the development of a more complete theoretical basis for understanding and deepening the psychology of tourism value, and the health behavior of seniors. Another important theoretical implication of this study is that it provides an empirical basis for expanding the understanding of senior tourism from a humanist geography perspective, as the psychological and behavioral elements of senior tourism cannot be separated from space or place, and their social and family roles. This study also explores the public health of senior tourism, and the meaning of “self-value” when seniors find value in travel, and improvement to their physical and mental health in transient mobility [47], as well as finding a pathway to “access” happiness. This process provides a basis for shaping the meaningful space of active aging and the impact of seniors’ travel health in a spatial context that can focus on happiness, and be reflected in a corresponding improvement in psychological health. This study provides the basis for the balance of self-value and tourism value. Based on this relationship, this study provides guidelines for the integration of tourism and public health issues in everyday spatial contexts from the perspective of active aging.

The practical contribution of this study is mainly at the level of physical and mental health promotion, from the perspective of positive aging; in particular, sensory perception, feelings, emotions, and cognition of senior travelers. Existing media communication channels and government public relations content output could encourage seniors to explore their own traits, to encourage positive psychology and behaviors that maximize their personal strengths, thereby enhancing the value of their tourism experiences. The findings may also be useful in developing strategies that are designed to convince the public of the advantages of supporting physical and mental health programs for seniors. Programs of this type can lead to increased recognition of the individual value of seniors, and the need to fund programs that support the building of positive relationships, and strengthen the transmission of positive psychological behaviors. This study also provides practical implications for the diversified development of the senior tourism market and recreation tourism, reflecting the pursuit of “self-fulfillment” values of seniors, positively reversing the image of senior public health groups, providing more possibilities for active aging, and serving as a model for the unaged [48]. The positive change in the image of the public health community will also have a positive effect on the communication effect of public health, highlighting the role of positive psychology and behavior, and changing the traditional and homogeneous perception of senior tourists in the existing market.

This study also has research limitations. Although the mixed research approach enabled the exploration of tourism value and health effects on seniors in some depth, the qualitative component of the study used a convenience sample, and the interviewees were all experienced in tourism, and had no underlying illnesses. This restricted sample was a major limitation. The narrative process of the positive aging of seniors in the interviews was also a limitation arising from differences in the narrative ability of older adults. The saturation of interview materials was another limitation. Future research could conduct additional dimensional screening of respondents, and a comparative study of the relationship between the presence or absence of underlying illness and frequency of travel on the value of travel and its health effects for seniors. An in-depth investigation through quantitative approaches to assess the tourism value indicators of active aging would also be worthwhile.

## Figures and Tables

**Table 1:** Descriptive statistical analysis.

Variables	2008–2018 Cohort		2011–2018 Cohort		2014–2018 Cohort	
	Tourists	Non-Tourists	Tourists	Non-Tourists	Tourists	Non-Tourists
Age (years)	78.55 (10.45)	86.95 (10.83)	79.03 (9.06)	85.39 (10.36)	78.92 (7.74)	84.88 (9.64)
Sex						
Female	278 (3.67)	7296 (96.33)	290 (6.89)	3922 (93.11)	141 (5.55)	2400 (94.45)
Male	317 (5.45)	5502 (94.55)	338 (9.20)	3334 (90.80)	187 (8.16)	2104 (91.84)
Education level						
No education	187 (2.22)	8244 (97.78)	206 (4.57)	4304 (95.43)	94 (3.48)	2605 (96.52)
Educated	408 (8.22)	4554 (91.78)	422 (12.51)	2952 (87.49)	234 (10.97)	1899 (89.03)
Type of residence						
Rural	227 (2.67)	8281 (97.33)	219 (5.36)	3864 (94.64)	132 (4.78)	2630 (95.22)
Urban	368 (7.53)	4517 (92.47)	409 (10.76)	3392 (89.24)	196 (9.47)	1874 (90.53)
Married or not						
No	251 (2.81)	8670 (97.19)	268 (5.61)	4508 (94.39)	209 (10.41)	1798 (89.59)
Yes	344 (7.69)	4128 (92.31)	360 (11.58)	2748 (88.42)	119 (4.21)	2706 (95.79)
Log annual household income	9.85 (1.24)	9.18 (1.48)	10.07 (1.43)	9.44 (1.76)	10.21 (1.24)	9.63 (1.60)
Chronically ill or not						
No	272 (3.79)	6911 (96.21)	205 (6.23)	3088 (93.77)	97 (4.85)	1901 (95.15)
Yes	323 (5.20)	5887 (94.80)	423 (9.21)	4168 (90.79)	231 (8.15)	2603 (91.85)
Disabled or not						
No	554 (5.19)	10,130 (94.81)	503 (9.41)	4843 (90.59)	253 (8.07)	2884 (91.93)
Yes	41 (1.51)	2668 (98.49)	125 (4.93)	2413 (95.07)	75 (4.42)	1620 (95.58)
Smoking or not						
No	466 (4.25)	10,503 (95.75)	487 (7.58)	5938 (92.42)	255 (6.33)	3774 (93.67)
Yes	129 (5.32)	2295 (94.68)	141 (9.66)	1318 (90.34)	73 (9.09)	730 (90.91)
Drink alcohol regularly or not						
No	458 (4.16)	10,541 (95.84)	482 (7.42)	6017 (92.58)	253 (6.23)	3807 (93.77)
Yes	137 (5.72)	2257 (94.28)	146 (10.54)	1239 (89.46)	75 (9.72)	697 (90.28)
Exercise regularly or not						
No	217 (2.24)	9480 (97.76)	230 (4.44)	4953 (95.56)	140 (3.93)	3426 (96.07)
Yes	378 (10.23)	3318 (89.77)	398 (14.74)	2303 (85.26)	188 (14.85)	1078 (85.15)

Note: Categorical variables report frequencies (percentages), and continuous variables report means (standard deviations).

**Table 2:** Effect of travel on mortality risk in elders.

		Death Risk		
		HR	95%CI	*p*
Model 1-1	2008–2018 cohort (*n* = 3393)			
	No outings	1.00		
	Outings	0.634 ***	0.551–0.728	<0.0001
Model 1-2	Number of trips	0.864 ***	0.808–0.925	<0.0001
Model 2-1	2011–2018 cohort (*n* = 7826)			
	No outings	1.00		
	Outings	0.640 ***	0.543–0.755	<0.0001
Model 2-2	Number of trips	0.893 ***	0.842–0.948	<0.0001
Model 3-1	2014–2018 cohort (*n* = 4814)			
	No outings	1.00		
	Outings	0.687 **	0.526–0.897	<0.01
Model 3-2	Number of trips	0.894 *	0.804–0.995	<0.05

Note: *** indicates significant at the 1 per 1000 level; ** indicates significant at the 1% level; * indicates significant at the 5% level; HR: hazard ratio.

## Data Availability

The study did not report any data.

## Appendix A. Interview Outline

### Warming Up

1. What is your current physical condition? Do you have any underlying conditions?
2. Have your trips changed before and after retirement? Why?
3. What is your usual travel pattern?

### Formal Interview

1. Please share a trip that you remember.

2. Can travel improve your health? What do you think about this issue?

3. What factors motivate you to travel? What do you think is the effect of travel on your health?

4. Please share a story about a trip that has had the greatest impact on you, including the cause, duration, and outcome of the story.

### Key Factors Interview

1. How do you think your personal strengths and traits have influenced your physical and mental health?

2. How do you think your personal expertise is reflected in your travel organization and travel experience?

3. What would your ideal senior life be like? What role does travel play in it?

## Appendix B

**Table A1:** Respondents’ Information.

Respondent No.	Age	Gender	Number of Trips (40–60)	Number of Trips (More than 60)
R1	65	Female	>10	>20
R2	66	Female	5–10	10–20
R3	69	Male	5–10	10–20
R4	65	Male	10–15	<10
R5	67	Female	20–30	>20
R6	73	Male	<10	<10
R7	72	Female	10–20	10–20
R8	65	Male	<5	10–20
